# Smoking is associated with elevated blood level of volatile organic compounds: a population-based analysis of NHANES 2017–2018

**DOI:** 10.1186/s13690-023-01070-x

**Published:** 2023-04-13

**Authors:** Guangjie Wu, Shiwei Gong, Yan He, Dong Liu

**Affiliations:** 1 Department of pharmacy, Tongji Hospital, Tongji Medical College, Huazhong University of Science and Technology, No.1095 Jiefang Avenue, Wuhan, Hubei provinve China; 2 School of pharmacy, Tongji Medical College, Huzhong University of Science and Technology, No.13 Hangkong Road, Jiefang Avenue, Wuhan, Hubei province China

**Keywords:** National Health and Nutrition Examination Survey, Smoking, Volatile organic compound, Blood concentration

## Abstract

**Background:**

The study aims to explore the association between cigarette smoking with blood exposure to volatile organic compounds using population data from the National Health and Nutrition Examination Survey (NHANES) 2017–2018.

**Methods:**

Based on the data of NHANES 2017–2018, we identified 1117 participants aged 18 to 65 years, who had complete VOCs testing data and filled out the Smoking-Cigarette Use and Volatile Toxicant questionnaires. The participants consisted of 214 dual-smoking persons, 41 E-cigarette smokers, 293 combustible-cigarette smokers and 569 non-smokers. We used One-way ANOVA and Welch’s ANOVA to compare differences of VOCs concentration among 4 groups and multivariable regression model to confirm the factors associated with VOCs concentration.

**Results:**

In dual-smoking and combustible-cigarette smokers, blood concentration of 2,5-Dimethylfuran, Benzene, Benzonitrile, Furan, Isobutyronitrile were higher than non-smokers. When compared with people who never smoked, E-cigarette smokers had similar blood concentrations of VOCs. Blood concentrations of Benzene, Furan, and Isobutyronitrile were significant higher in combustible-cigarette smokers than in E-cigarette smokers. In the multivariable regression model, dual-smoking and combustible-cigarette smoking were associated with elevated blood concentrations of several VOCs except 1,4-Dichlorobenzene, while E-cigarette smoking was only associated with elevated 2,5-Dimethylfuran concentration.

**Conclusions:**

Smoking, mainly dual-smoking and combustible-cigarette smoking, is associated with elevated blood concentration of VOCs, while the effect is weak in E-cigarette smoking.

**Supplementary Information:**

The online version contains supplementary material available at 10.1186/s13690-023-01070-x.

## Background

Globally, nearly one in every four adults smokes [1]. Smoking is thought to be concerned with several disease, including cancers, cardiovascular diseases, respiratory disorders and reproductive diseases [2]. More than 7000 chemicals with different toxicities have been identified in tobacco smoke [3]. The relationship between smoking-induced diseases and these toxic ingredients has not been fully understood. Although nicotine has been widely recognized as the primary addictive substance of smoking with potential deleterious effects, non-nicotine toxicities such as volatile organic compounds (VOCs) are more strongly associated with morbidity and mortality of smoking [4]. VOCs are organic chemical compounds that easily reach in the environment under normal conditions and found in various products such as tobacco smoke, chlorinated water, perfumes, paint removers, adhesives, new clothing, plastics or kerosene heaters [5]. Also, people can be exposed to VOCs through daily activities, including drinking, eating, bathing, or swimming [6]. People who inhale VOCs can develop different kinds of diseases. The impact of VOCs on respiratory tract has always been the focus of research. For example, propylene glycol, benzene and formaldehyde have high reactivity on the epithelial lining and mucous membrane of respiratory tract, which result in airway inflammation causing respiratory disorders [7]. Besides, more than 10 VOCs such as acetaldehyde, acetone, ethylbenzene, o-Xylene, could causing respiratory effects [8]. In a meta-analysis by Kyle L. Alford, asthma, wheezing, throat irritation are the most common symptom related to VOCs exposure [7]. In addition to their effects on the respiratory system, VOCs are also thought to be associated with cardiovascular and nervous system dysfunction, evidenced by animal experiments or human exposure reviews. These VOCs include acetone, benzene, m/p/o-xylene, tetrachlorocarbon, toluene, trichloroethylene and 2-Methylbutane [8]. Carcinogenic effect is also an important topic of VOCs. The World Health Organization (WHO) classified several VOCs into different kinds of carcinogens in 2010. Formaldehyde, benzene and trichloroethylene have been classified into Group 1 as “known human carcinogens”, while styrene and tetrachloroethylene have been classified into Group 2 A and 2B as “possible or probable human carcinogens” [9].

So far, a few researches have explored the relationship between smoking and VOCs exposure in human [10–12]. In a two-arm counterbalanced, crossover study of 36 healthy dual users of electronic-cigarette (e-cigarette) and combustible cigarettes, Helen Gideon and colleagues found that urinary concentrations of VOCs metabolites were higher during smoking combustible cigarettes compared to vaping e-cigarette. The fold-difference in concentrations when smoking relative to vaping ranged from 1.31 to 7.09 according to different VOCs [10]. In a large population-based research - Population Assessment of Tobacco and Health Study (PATH Study) conducted from 2013 to 2014, De Jesús Víctor and colleagues found urinary acrolein, crotonaldehyde, isoprene, acrylonitrile, and 1,3-butadiene were significantly higher in smokers than in non-smokers, while small differences of VOCs were found between e-cigarette users and non-smokers [11]. In the same study, analysis focused on smokeless tobaccos (SLT) users, showed that VOC biomarker concentrations were similar for exclusive SLT users and never tobacco users [12]. All three studies focused on urinary samples when measuring VOCs. However, since VOCs contain many kinds of compounds, of which the metabolic pathway is complex, the distribution of VOCs in human body is quite important. Valentina Longo and colleagues analyzed samples from urine, blood and human semen and found that only 12 of 135 VOCs presented in all 3 specimens, which meant that a significant proportion of VOCs were found only in urine, blood or human semen respectively [13]. Researchers have found that VOCs concentration could be used for diagnostic application of several diseases [14, 15], while it quite depends on the sample sources. According to Deng et al., only hexanal and heptanal from the blood could be used as potential lung cancer biomarkers [16]. Based on this result, we could infer that VOCs in different body fluids have different diagnostic values. So, it is not comprehensive to only know the data of urinary VOCs concentration in smoking persons for the purpose of evaluation on the association between smoking and VOCs exposure.

Given these facts, we conduct this research based on the data from the National Health and Nutrition Examination Survey 2017–2018 (NHANES) to compare blood VOCs concentration in dual-smoking, e-cigarette smoking, combustible-cigarette smoking and non-smokers.

## Methods

Data were extracted from the NHANES 2017-18 dataset with Rstudio (version 4.2.0). The NHANES is a population-based, cross-sectional survey to characterize health status of non-institutionalized individuals in the U.S, conducted by National Center for Health Statistics of the Centers for Disease Control and Prevention. Data of the NHAENS is released in 2-year cycles and utilizes a multistage probability sampling design to create a nationally representative sample for each cycle. All participants provide written informed consent before completing the survey [17]. Participants in the NHANES 2017–2018 database were included in this study if they completed both the Smoking-Cigarette Use (SMQ_J) and Volatile Toxicant (VTQ_J) questionnaires and were 18 to 65 years old. The Questionnaire SMQ_J provides a history of cigarette use, age at initiation, past 30-day use, cigarette brand, sub-brand and other related details. Questionnaire VTQ_J includes data about the participants home, activities, amount of time spent in various locations and exposure to different chemicals over the past 48 h. VTQ_J involves 12 scenarios, including owing a house with a garage, storing paints or fuels inside home, use of moth balls, inhalation of smoke, cooking with natural gas, pumping gas into a car, spending time in pool/hot tub/steam room, use of dry cleaning solvent, bathing, breathing paint fumes, breathing diesel fumes, and breathing fingernail polish fumes. All subjects with “Refuse”, “Don’t know” or missing answers to any question on either questionnaires, were removed. Participants were divided into 4 groups (Dual Smoking, E-cigarette, Combustible-Cigarette and Non-Smoking) according to the answers of question SMQ890 (Have you ever smoked a regular cigar, cigarillo or little filtered cigar even one time?) and question SMQ900 (Have you ever used an e-cigarette even one time?). Blood VOCs data was extracted from VOC laboratory data (VOCWB_J). In NHANES 2017–2018, for VOCs measurement, an automated analytical method was developed using capillary gas chromatography and mass spectrometry with selected-ion monitoring detection and isotope-dilution. This method quantifies levels of individual VOCs in whole blood to low-parts-per-trillion range. This method is applicable for determining these quantities and investigating cases of sustained or recent low-level exposure. In this part, each value of VOC concentration was compared with a specific detection limit and recorded with a qualitative judgment as “at or above detection limit” or “below lower detection limit”. The lower detection limit of 40 tested VOCs were shown in the codebook of VOCWB_J. Any tested VOCs displayed “all below lower detection limit” were considered to have no differences among the groups.

### Statistical analysis

Statistical analysis were performed using SPSS software (version 21). Kolmogorov-Smirnova test was used to test the normality of VOCs concentration data, and Levene test was used to detect the homogeneity of variance. All the VOCs concentration data showed non-normal distribution. One-way ANOVA and Welch’s ANOVA were used to compare differences of VOCs concentration among 4 groups according to the homogeneity of variance as described before [18]. Games-Howell test was used for post-hoc pairwise comparisons when heterogeneity of variance existed [18]. Multivariable linear regression analysis were conducted for VOCs that showed differences among the 4 groups to find out factors associated with the different concentrations. When performing the multivariable linear regression analysis, we also included answers to questionnaire VTQ_J as confounding factors. This questionnaire included data about the participants’ home, activities, amount of time spent in various locations and exposure to different chemicals over t0f1che past 48 hours of the surveying day. In VTQ_J, participants were asked “Does your home have an attached garage?”, or “In the last three days, did you inhale smoke from any source for 10 or more minutes?”, totally 12 questions, and answering any of these questions with a “yes” was marked as “having access to VOCs from any source”. In the regression model, sex, age, having access to VOCs, s well as smoking patterns were included as covariates. For covariate sex, the reference value was “male”, for covariate “having access to VOCs from any source”, the reference value was”Yes”, and for covariate “use patterns”, the reference value was “not this pattern”. All analyses were considered significant at two-tailed p-values of < 0.05.

## Results

### Characteristics of the Sample, NHANES 2017–2018

There were 1117 participants included in the analysis. 214 were dual-smoking, 41 reported smoking only E-cigarette, 293 reported smoking only combustible-Cigarette, and the rest 569 were non-smokers. Detail demographic information of the included participants is shown in Table 1.

**Table 1:** Characteristics of the sample population, NHANES 2017–2018

	Dual Smoking (n = 214)	E-Cigarette (n = 41)	Combustible- Cigarette (n = 293)	Non- Smoking (n = 569)
**Sex**				
Male	135(24.8)	15(2.8)	203(37.2)	192(35.2)
Female	79(13.8)	26(4.5)	90(15.7)	377(65.9)
**Age**				
18 ~ 40	139(26.0)	38(7.1)	103(19.3)	254(47.6)
41 ~ 65	75(12.9)	3(0.5)	190(32.6)	315(54.0)
**Race**				
Mexican American	25(14.5)	6(3.5)	38(22.1)	103(59.9)
Other Hispanic	13(12.3)	2(1.9)	29(27.4)	62(58.5)
Non-Hispanic White	105(29.9)	9(2.6)	103(29.3)	134(38.2)
Non-Hispanic Black	33(12.6)	11(4.2)	85(32.4)	133(50.8)
Non-Hispanic Asian	18(11.2)	10(6.2)	19(11.8)	114(70.8)
Other Race- Including Multi-Racial	20(30.8)	3(4.6)	19(29.2)	23(35.4)
**Education level**				
Less than 9th grade	5(8.2)	2(3.3)	11(18.0)	43(70.5)
9-11th grade	27(27.0)	2(2.0)	26(26.0)	45(45.0)
High school graduate or General Equivalency Diploma	58(21.5)	6(2.2)	84(31.1)	122(45.2)
Some college or AA degree	81(23.6)	12(3.5)	92(26.8)	158(46.1)
College graduate or above	29(11.0)	8(3.0)	75(28.5)	151(57.4)
Missing	14(17.5)	11(13.8)	5(6.3)	50(62.5)
**Ratio of family income to poverty**				
<5	166(21.2)	29(3.7)	198(25.3)	389(49.7)
≥ 5	23(12.4)	6(3.2)	62(33.5)	94(50.8)
Missing	25(16.7)	6(4.0)	33(22.0)	86(57.3)

Note: Data were shown as number (row percentage)

### Differences of blood VOCs among the sample population

31 of 40 tested VOCs were found with no differences in blood concentration among the 4 groups, including 9 with a result of “All below lower detection limit”. While 9 VOCs were statistically different among the groups. These 9 VOCs were 2,5-Dimethylfuran, Heptane, Benzene, Benzonitrile, 1,4-Dichlorobenzene, Ethyl Acetate, Furan, Isobutyronitrile, Methylene Chloride, as shown in Table 2.

**Table 2:** Overall differences of blood VOCs among the sample population

VOCs	F value	*p*
2,5-Dimethylfuran	40.675	*p* < 0.001
Heptane	257.023	*p* < 0.001
Benzene	32.055	*p* < 0.001
Benzonitrile	8.027	*p* < 0.001
1,4-Dichlorobenzene	6.051	*p* = 0.001
Ethyl Acetate	3.418	*p* = 0.017
Furan	36.139	*p* < 0.001
Isobutyronitrile	14.436	*p* < 0.001
Methylene Chloride	32.581	*p* < 0.001
Octane	0.421	*p* = 0.738
1,2-Dichloroethane	0.560	*p* = 0.641
Tetrachloroethene	0.244	*p* = 0.866
Bromoform	0.434	*p* = 0.728
Cyclohexane	0.513	*p* = 0.673
Chloroform	0.205	*p* = 0.893
Dibromochloromethane	0.903	*p* = 0.439
Ethylbenzene	0.134	*p* = 0.940
Chloroethane	0.294	*p* = 0.830
Isopropylbenzene	0.313	*p* = 0.816
MTBE	0.212	*p* = 0.888
o-Xylene	0.297	*p* = 0.828
Trichloroethene	0.192	*p* = 0.902
1,1,1-Trichloroethane	0.294	*p* = 0.830
Tetrahydrofuran	0.122	*p* = 0.947
m-/p-Xylene	0.203	*p* = 0.894
Hexane	0.992	*p* = 0.398
Bromodichloromethane	0.893	*p* = 0.446
Chlorobenzene	0.662	*p* = 0.576
Carbon Tetrachloride	0.663	*p* = 0.576
Methylcyclopentane	0.739	*p* = 0.530
Methyl Isobutyl Ketone	0.997	*p* = 0.394

Note: Concentrations of 1,1,1,2-Tetrachloroethane, 1,2-Dichlorobenzene, 1,3-Dichlorobenzene, 1,2-Dibromoethane, Diethyl Ether, Nitrobenzene, aaa-Trifluorotoluene, 1,2,3-Trichloropropane, and Vinyl Bromide were all below lower detection limit and were not shown in Table 2

### Comparison of blood VOCs concentration among different smoking types

In order to find out the effects of different smoking types on blood VOCs, we conducted a pairwise comparison process. As shown in Supplementary Tables 1, people who were dual-smoking had a higher blood level of 2,5-Dimethylfuran, Benzene, Benzonitrile, 1,4-Dichlorobenzene, Furan, Isobutyronitrile than people who never smoked. When compared with people who never smoked, E-cigarette smokers had similar blood concentrations of the 9 VOCs, while combustible-smoking resulted in higher blood concentration of 2,5-Dimethylfuran, Benzene, Benzonitrile, Furan, and Isobutyronitrile. As for the effects of two common kinds of cigarettes, blood concentration of Benzene, Furan, Isobutyronitrile were significant higher in combustible cigarette smokers than in E-cigarette smokers. Details are shown in Supplementary Table 1.

### Factors associated with blood concentration of VOCs

Multivariable linear regression was conducted to find out the factors that influenced the blood concentration of the 6 VOCs among the 4 groups. Dual-smoking and combustible-cigarette smoking were associated with elevated blood concentration of 5 VOCs except 1,4-Dichlorobenzene, which was associated with smokers’ age only. While E-cigarette smoking was only associated with elevated 2,5-Dimethylfuran concentration. Besides smoking types, sex and age also accounted for the higher concentration of several VOCs to some extent. Decreasing concentration of Benzonitrile was found in female sex, while this effect was not seen in other VOCs. Increasing age was also associated with elevated concentration of 2,5-Dimethylfuran, Benzene, Furan and 1,4-Dichlorobenzene which was mentioned above. Results are shown in Table 3.

**Table 3:** Multivariable linear regression model of 6 VOCs

variate	2,5-Dimethylfuran			Benzene			Benzonitrile			1,4-Dichlorobenzene			Furan			Isobutyronitrile		
	β	95%CI	p	β	95%CI	p	β	95%CI	p	β	95%CI	p	β	95%CI	p	β	95%CI	p
Intercept	0.004	−0.022 ~ 0.030	0.760	0.017	−0.034 ~ 0.068	0.522	0.113	0.084 ~ 0.143	< 0.001	0.521	−0.844 ~ 1.885	0.454	0.015	0.004 ~ 0.027	0.010	0.022	0.007 ~ 0.037	0.005
Sex	-0.001	−0.009 ~ 0.008	0.881	-0.007	−0.024 ~ 0.009	0.405	-0.010	−0.020 ~−0.001	0.034	-0.168	−0.608 ~ 0.273	0.456	0.000	−0.003 ~ 0.004	0.864	-0.002	−0.007 ~ 0.003	0.439
Age	0.000	0.000 ~ 0.001	0.011	0.001	0.000 ~ 0.002	< 0.001	0.000	0.000 ~ 0.001	0.084	0.022	0.007 ~ 0.037	0.005	0.000	0.000 ~ 0.000	0.001	0.000	0.000 ~ 0.000	0.345
Dual-smoking	0.057	0.046 ~ 0.068	< 0.001	0.109	0.088 ~ 0.131	< 0.001	0.019	0.007 ~ 0.031	0.003	-0.503	−1.082 ~ 0.076	0.089	0.029	0.024 ~ 0.034	< 0.001	0.015	0.009 ~ 0.022	< 0.001
E-Cigarette	0.023	0.001 ~ 0.044	0.042	0.026	−0.018 ~ 0.069	0.248	0.009	−0.016 ~ 0.034	0.476	0.820	−0.337 ~ 1.978	0.165	0.005	−0.004 ~ 0.015	0.281	0.004	−0.009 ~ 0.017	0.586
Combustible- smoking	0.030	0.020 ~ 0.040	< 0.001	0.051	0.031 ~ 0.070	< 0.001	0.021	0.010 ~ 0.033	< 0.001	-0.183	−0.710 ~ 0.344	0.496	0.013	0.009 ~ 0.017	< 0.001	0.014	0.008 ~ 0.020	< 0.001
Having access to VOCs from any source	-0.009	−0.026 ~ 0.007	0.265	-0.190	−0.052 ~ 0.014	0.254	0.003	−0.016 ~ 0.022	0.736	-0.393	−1.273 ~ 0.487	0.381	-0.005	−0.013 ~ 0.002	0.165	0.006	−0.004 ~ 0.016	0.243

β: Partial regression coefficient

## Discussion

In this real-world cross sectional study, we found that in dual-smoking and combustible-cigarette smoking persons blood exposure to several VOCs were higher than non-smokers, while this effect was not seen in E-cigarette only smokers. When comparing combustible-cigarette and E-cigarettes smoking, blood concentrations of several VOCs (Benzene, Furan, Isobutyronitrile) were significant higher in combustible cigarette smokers than in E-cigarette smokers. In the multivariable linear regression model including sex, age, and access to VOCs from any source as confounding factors, smoking types still had the strongest positive relationship with elevated blood concentration of VOCs. In the regression model, E-cigarette smoking was only associated with elevated concentration of 2,5-Dimethylfuran. Though statistically significant, age had relatively small influence on blood concentration of VOCs, but was the only factor that influenced the concentration of 1,4-Dichlorobenzene. We also found female sex was associated with decreasing concentration of Benzonitrile, which was not found in other VOCs.

In the previous study, VOCs has been quantified in 50 U.S brand cigarettes smoke showing that smoking increases VOCs exposure up to 6-fold [19]. However, total VOCs emission of cigarette smoke was often tested under experiment conditions such as ISO 3308 and Canadian Intense (CI) smoking protocols, not in human beings [19, 20]. Human exposure to VOCs were often tested using urine sample. In an active smoking experiments conducted in 36 healthy people by Gideon St.Helen and colleagues, concentrations of urinary VOCs metabolites were higher during smoking compared to non-smokers, the fold-difference of different VOCs ranged from 1.31 to 7.09 [10]. These results supported our findings. Whereas, the study also found higher Benzene concentration when comparing E-cigarettes vaping with abstention which shows a similar trend in our study. The reason may be attribute to different power of the vaping devices and the specific types of E-cigarette, since Benzene has been detected in some re-fill e-liquid or cartridges [21] and associated with devices’ working-temperature [22]. However, the sample size of the study was quite small and not a population-level research. In a large scale cohort study focused on tobacco use and its health effects, De Jesús Víctor R and colleagues found that combustible-cigarette smokers showed higher urinary VOCs metabolites concentrations than E-cigarette smokers, and non-smokers, while small differences were observed when comparing E-cigarette smokers and non-smokers [11]. These results from urine sample were consistent with ours from blood sample.

It seems that E-cigarette is “healthier” than combustible cigarette as E-cigarette smokers have lower systemic exposure to toxicities compared to combustible-cigarette smokers [22], but concerns about its potential harm to public health still exist [23]. Comparisons of VOCs biomarkers between E-cigarette smokers and combustible-cigarette smokers have been reported. In the PATH study and a cross-sectional study conducted in UK, the two research groups all found that E-cigarette smokers had lower VOCs biomarkers than combustible-cigarette smokers [24, 25]. Recent studies have shown that E-cigarette smokers had stronger knowledge of cigarette-related harm than dual-smoking and combustible-cigarette smokers, which would influence smoking behaviors and thus could partially explain the lower concentration of VOCs in E-cigarette smokers [26, 27]. As mentioned above, these studies also used urine samples for measuring VOCs biomarkers. In our study, blood concentration of Benzene, Furan, Isobutyronitrile were significant lower in E-cigarette smokers than in combustible-cigarette smokers. In a sense, the result of our study is complementary to previous studies, which confirmed that the trend of urine as well as blood VOCs concentrations between E-cigarette smokers and combustible-cigarette smokers are consistent. Though concentration of VOCs is lower in E-cigarette smokers, we couldn’t conclude that E-cigarette smokers are at less risks of potential harm, because toxicities are not only depended on VOCs.

In the multivariable regression model, smoking types was found to be a factor that influenced VOCs level as expected. Dual-smoking and combustible-cigarette smoking both had strong association with elevated VOCs blood level except 1,4-Dichlorobenzene. In fact, the relationship of smoking and exposure to 1,4-Dichlorobenzene was not consistent in previous studies. Ram B. Jain reported that in the U.S adults (≥ 20 years), non-smokers had higher blood concentration of 1,4-Dichlorobenze than smokers (0.083 vs. 0.064 ng/ml, p < 0.01), while the other 6 VOCs were contrary [28]. Another research by Lin and colleagues showed no association of smoking with the air and blood concentration of 1,4-Dichlorobenzene [28]. The reason of these inconsistent results may be due to that 1,4-Dichlorobenzene is a common component of moth repellents and deodorizers, but not from cigarettes [29–31]. In our study, exposure to moth repellents and deodorizers was categorized into “have access to VOCs from any source” as confounding factors, which showed no statistical significance for 1,4-Dichlorobenzene among different groups. This could be explained by the complexity of the confounding factors since it consists of 12 different questions. On the other hand, we found age was the only factor that influenced the concentration of 1,4-Dichlorobenzene. As far as we know, the relationship between age and exposure to 1,4-Dichlorobenzene has not been reported, therefore the specific mechanism is unclear. This might be partially explained by the association between age and exposure to moth repellents or deodorizers, thus leading to different exposure to 1,4-Dichlorobenzene. Further research is needed to explore the clear association between smoking and 1,4-Dichlorobenzene exposure under different situations. We also found female sex was associated with decreasing concentration of Benzonitrile. This difference of the Benzonitrile level in females may be explained by metabolic difference between males and females, as described by De Jesús Víctor [11].

There are some important limitations to our study. First, the smoking patterns were self-reported, which may not reflect the actual use patterns. Another limitation of this study is that, information on the types of combustible cigarettes and E-cigarettes was not analyzed, and it is not clear whether this has an impact on blood VOCs exposure since toxicity contents may depend on specific types of combustible cigarettes and E-cigarettes [21]. More work is needed to overcome these limitations.

In conclusion, in this population-based cross sectional study, we confirm that dual-smoking and combustible-cigarette smoking are associated with higher blood concentrations of several VOCs than non-smokers. By contrast, this effect of E-cigarette smoking is absent for most VOCs.

## Electronic supplementary material

Below is the link to the electronic supplementary material.

Supplementary Material 1

## Data Availability

The data underlying this article are available in NHANES 2017–2018 at www.cdc.gov/nchs/nhanes/index.htm, and can be accessed without a specific account.

## List of abbreviations

NHANES: National Health and Nutrition Examination Survey
VOCs: Volatile organic compounds
MTBE: Methyl tert-butyl ether
