# Supplementary material for: Smoking is associated with elevated blood level of volatile organic compounds: a population-based analysis of NHANES 2017–2018
Source: Arch Public Health. 2023 Apr 13;81:55. doi: 10.1186/s13690-023-01070-x (PMC10103525; doi:10.1186/s13690-023-01070-x)
Supplement: Supplementary file 1 — Supplementary Material 1 [file 13690_2023_1070_MOESM1_ESM.docx]

Supplementary Table 1. Pairwise comparison of blood VOCs among different smoking population

| VOCs | Group A | Group B | Difference of Mean Value  (A-B) | SE | 95% CI | | *p* |
| --- | --- | --- | --- | --- | --- | --- | --- |
|  |  |  |  |  | Lower limit | Upper limit |  |
| 2,5-  Dimethylfuran | Dual-  Smoking | E-Cigarette | 0.0389 | 0.018 | -0.009 | 0.087 | *p* = 0.154 |
|  |  | Combustible-  Cigarette | 0.0241 | 0.008 | 0.003 | 0.045 | *p* = 0.019 |
|  |  | Non-  Smoking | 0.0556 | 0.006 | 0.041 | 0.070 | *p* < 0.001 |
|  | E-Cigarette | Combustible-  Cigarette | -0.0148 | 0.018 | -0.063 | 0.034 | *p* = 0.848 |
|  |  | Non-  Smoking | 0.0167 | 0.017 | -0.029 | 0.063 | *p* = 0.767 |
|  | Combustible-  Cigarette | Non-  Smoking | 0.0315 | 0.006 | 0.016 | 0.047 | *p* < 0.001 |
| Heptane | Dual-  Smoking | E-Cigarette | 0.0000 | 0.000 | 0.000 | 0.000 | *-* |
|  |  | Combustible-  Cigarette | -0.0045 | 0.003 | -0.013 | 0.004 | *p* = 0.528 |
|  |  | Non-  Smoking | -0.0001 | 0.000 | 0.000 | 0.000 | *p* = 0.749 |
|  | E-Cigarette | Combustible-  Cigarette | -0.0045 | 0.003 | -0.013 | 0.004 | *p* = 0.528 |
|  |  | Non-  Smoking | -0.0001 | 0.000 | 0.000 | 0.000 | *p* = 0.749 |
|  | Combustible-  Cigarette | Non-  Smoking | 0.0044 | 0.003 | -0.004 | 0.013 | *p* = 0.542 |
| Benzene | Dual-  Smoking | E-Cigarette | 0.0977 | 0.016 | 0.057 | 0.139 | *p* < 0.001 |
|  |  | Combustible-  Cigarette | 0.0486 | 0.015 | 0.011 | 0.086 | *p* = 0.005 |
|  |  | Non-  Smoking | 0.1063 | 0.012 | 0.075 | 0.137 | *p* < 0.001 |
|  | E-Cigarette | Combustible-  Cigarette | -0.0492 | 0.015 | -0.087 | -0.011 | *p* = 0.006 |
|  |  | Non-  Smoking | 0.0085 | 0.012 | -0.023 | 0.040 | *p* = 0.891 |
|  | Combustible-  Cigarette | Non-  Smoking | 0.0577 | 0.010 | 0.031 | 0.084 | *p* < 0.001 |
| Benzonitrile | Dual-  Smoking | E-Cigarette | 0.0164 | 0.010 | -0.009 | 0.042 | *p* = 0.349 |
|  |  | Combustible-  Cigarette | -0.0056 | 0.008 | -0.026 | 0.015 | *p* = 0.898 |
|  |  | Non-  Smoking | 0.0203 | 0.006 | 0.005 | 0.035 | *p* = 0.003 |
|  | E-Cigarette | Combustible-  Cigarette | -0.0220 | 0.010 | -0.049 | 0.005 | *p* = 0.153 |
|  |  | Non-  Smoking | 0.0039 | 0.009 | -0.019 | 0.027 | *p* = 0.969 |
|  | Combustible-  Cigarette | Non-  Smoking | 0.0259 | 0.007 | 0.009 | 0.043 | *p* = 0.001 |
| 1,4-  Dichlorobenzene | Dual-  Smoking | E-Cigarette | -1.0279 | 1.024 | -3.773 | 1.717 | *p* = 0.748 |
|  |  | Combustible-  Cigarette | -0.5363 | 0.231 | -1.134 | 0.061 | *p* = 0.096 |
|  |  | Non-  Smoking | -0.5652 | 0.160 | -0.976 | -0.154 | *p* = 0.002 |
|  | E-Cigarette | Combustible-  Cigarette | 0.4915 | 1.049 | -2.309 | 3.292 | *p* = 0.966 |
|  |  | Non-  Smoking | 0.4626 | 1.035 | -2.307 | 3.232 | *p* = 0.970 |
|  | Combustible-  Cigarette | Non-  Smoking | -0.0289 | 0.276 | -0.741 | 0.683 | *p* = 1.000 |
| Ethyl Acetate | Dual-  Smoking | E-Cigarette | 0.3480 | 0.244 | -0.283 | 0.979 | *p* = 0.483 |
|  |  | Combustible-  Cigarette | -0.4269 | 0.420 | -1.509 | 0.655 | *p* = 0.739 |
|  |  | Non-  Smoking | 0.3286 | 0.244 | -0.303 | 0.960 | *p* = 0.534 |
|  | E-Cigarette | Combustible-  Cigarette | -0.7749 | 0.342 | -1.658 | 0.108 | *p* = 0.108 |
|  |  | Non-  Smoking | -0.0195 | 0.011 | -0.048 | 0.009 | *p* = 0.294 |
|  | Combustible-  Cigarette | Non-  Smoking | 0.7555 | 0.342 | -0.128 | 1.639 | *p* = 0.123 |
| Furan | Dual-  Smoking | E-Cigarette | 0.0261 | 0.004 | 0.015 | 0.037 | *p* < 0.001 |
|  |  | Combustible-  Cigarette | 0.0143 | 0.004 | 0.004 | 0.025 | *p* = 0.002 |
|  |  | Non-  Smoking | 0.0282 | 0.003 | 0.020 | 0.036 | *p* < 0.001 |
|  | E-Cigarette | Combustible-  Cigarette | -0.0118 | 0.004 | -0.021 | -0.002 | *p* = 0.010 |
|  |  | Non-  Smoking | 0.0020 | 0.003 | -0.006 | 0.010 | *p* = 0.893 |
|  | Combustible-  Cigarette | Non-  Smoking | 0.0138 | 0.002 | 0.008 | 0.020 | *p* < 0.001 |
| Isobutyronitrile | Dual-  Smoking | E-Cigarette | 0.0135 | 0.004 | 0.004 | 0.023 | *p* = 0.001 |
|  |  | Combustible-  Cigarette | 0.0003 | 0.005 | -0.012 | 0.013 | *p* = 1.000 |
|  |  | Non-  Smoking | 0.0152 | 0.003 | 0.008 | 0.022 | *p* < 0.001 |
|  | E-Cigarette | Combustible-  Cigarette | -0.0132 | 0.005 | -0.025 | -0.001 | *p* = 0.021 |
|  |  | Non-  Smoking | 0.0018 | 0.002 | -0.004 | 0.008 | *p* = 0.848 |
|  | Combustible-  Cigarette | Non-  Smoking | 0.0149 | 0.004 | 0.005 | 0.025 | *p* = 0.001 |
| Methylene Chloride | Dual-  Smoking | E-Cigarette | 0.0000 | 0.000 | 0.000 | 0.000 | *-* |
|  |  | Combustible-  Cigarette | -0.0076 | 0.005 | -0.022 | 0.007 | *p* = 0.513 |
|  |  | Non-  Smoking | -0.0002 | 0.000 | -0.001 | 0.000 | *p* = 0.749 |
|  | E-Cigarette | Combustible-  Cigarette | -0.0076 | 0.005 | -0.022 | 0.007 | *p* = 0.513 |
|  |  | Non-  Smoking | -0.0002 | 0.000 | -0.001 | 0.000 | *p* = 0.749 |
|  | Combustible-  Cigarette | Non-  Smoking | 0.0074 | 0.005 | -0.007 | 0.022 | *p* = 0.538 |
